# Active ageing: process and determinants among middle-aged men in rural and urban communities in Lagos State, Nigeria

**DOI:** 10.11604/pamj.2021.39.195.27546

**Published:** 2021-07-13

**Authors:** Augustine Ajogwu, Kofo Odeyemi

**Affiliations:** 1 Department of Community Health and Primary Health Care, Lagos University Teaching Hospital, PMB 12003, Idi-Araba, Surulere, Lagos, Nigeria,; 2 Department of Community Health and Primary Care, College of Medicine University of Lagos/ Lagos University Teaching Hospital, PMB 12003,Idi-Araba, Surulere, Lagos, Nigeria

**Keywords:** Active ageing processes, quality of life, Africa

## Abstract

**Introduction:**

active ageing is the process of optimizing opportunities for health in order to enhance quality of life and wellbeing. It is influenced by physical activity, social participation and social network, cognitive and continuous learning and socio-economic factors. It involves disease prevention and promotion of healthy behaviours that can reduce the risk and occurrence of non-communicable diseases in middle age and also at old age. The study aimed to determine and compare the active ageing process and its determinants among middle-aged men in rural and urban areas in Nigeria.

**Methods:**

this was a comparative cross-sectional study among middle-aged men 40-60 years using mixed methods. A multi-stage sampling technique was used to select 720 respondents. A structured interviewer administered questionnaire and Key informant interviews were used to collect data.

**Results:**

respondents in the rural area were a little older compared to the respondents in the urban area with a mean age of 49.6 ± 6.3 years and 48.6 ± 6.2 years respectively. A higher proportion of the respondents in the rural area (83.2%) than the respondents in the urban area (73.8%) practice good active ageing processes in their lives. There was a significant association between education of respondent and the practice of active ageing among respondents in the urban and rural areas. Multivariate logistic regression showed that physical activity (aOR 7.62, 95% CI: 243-23.94, P = 0.001), life-long learning (aOR 51.73, 95% CI: 12.14-220.49, P = 0.000) and community participation (aOR 3.46, 95% CI: 2.51-4.77, P=0.000) are predictors of active ageing.

**Conclusion:**

the study showed that respondents in the rural area practice good active ageing compared to the respondents in the urban area and hence engaged more in sufficient active life in their daily activities, reducing the risk of non-communicable diseases.

## Introduction

Active ageing is the process of optimizing opportunities for health, participation and security in order to enhance quality of life and wellbeing [1]. The World Health Organization (WHO) argues that countries can afford to get old if governments, international organizations and civil society adopt active ageing policies and programmes that enhance the health, participation and security of individual citizens [1]. According to World Health Organization, 60% of all deaths globally are attributed to non-communicable diseases and 80% of these are in low/middle-income countries. Active ageing policies and programmes are needed to enable people to continue to work according to their capacities and preferences as they grow older and to prevent or delay disabilities and chronic diseases that are costly to individuals, families and the health care system [2]. It is therefore, crucial to ensure that citizens have every opportunity to remain active. Active ageing goal is to shift focus from clinical model to a health promotion model of care and should serve as a bench mark for policies for all age groups as well as individuals for disease prevention and promotion of healthy behaviours in a supportive environment [1].

Active ageing allows people to realize their potential for physical, social and mental wellbeing throughout the life course and to participate in society while providing them with adequate protection, security and care when they need it. It refers to continuing participation in social, economic, cultural, spiritual and civic affairs not just the ability to be physically active or to participate in the labour force. It aims to extend healthy life expectancy and quality of life for all people as they age [3]. Studies have shown that social participation, regular physical activity and continuous learning activity reduces the risk of cardiac death by 20% to 25% among people with established heart disease, reduces coronary heart disease by 10% and lowers the risk of stroke by 20%, reduces the risk of type 2 diabetes mellitus by 33-50%, colon cancer risk by 40-50%, lower the risk of breast cancer and osteoporosis by 30% and 50% respectively. Also, men has shown to develop NCDs earlier than women. [1, 4-6] The objective of the study was to determine and compare the active ageing processes and their determinants among a population of middle-aged men in rural and urban communities to improve the quality of life.

## Methods

**Study design and setting:** the study was a mixed method, comparative cross sectional study, using a mixture of quantitative and qualitative study design. This was to get in-depth characteristics of the population assessed. The study was conducted in Surulere (urban) and Ikorodu (rural) local government areas of Lagos state, located in the southwest geopolitical zone in Nigeria.

**Study population:** the population consisted of middle-aged men (40 to 60 years old). Men, who reside in the community for more than one year.

**Sampling technique:** the sample size was calculated using the standard formula for Comparative study. Based on previous studies, 92.1% urban respondents participate actively in community activities while 84.5% of rural respondents participate actively in community activities. This was used to calculate the sample size at 95% confidence interval, alpha of 0.05 and expected non response rate of 10%. A sample size of 360 for urban and 360 for rural, making a total of 720 was used for the study. Multi- stage sampling technique was used to select respondents.

**Study instrument:** the tool for quantitative data was a structured pre-tested interviewer-administered questionnaire. The questionnaire was adapted from previous studies [7-11]. The questionnaire consisted of six sections. Section A; collected information on socio-demographic characteristics. Section B; was questions on their knowledge of active ageing processes and its determinants. Section C; collected information on level of physical activity which consisted their forms of physical activity, at work, travel to and from places, recreational activities and sedentary behaviour. It also accessed the duration and frequency of each activity per week. Section D; collected information on respondents participation in the community activities and questions dwelt on their level of participation in community activities, leisure and social networking. Section E; consisted of information on life-long learning process which were on level of acquiring new skills, learning and improvement on the job and Section F; consisted of information on factors that affect the active ageing process and its determinants. The qualitative data was by means of key informant interviews of the opinion leaders in the communities using an interview guide. Section A was on socio-demographics of the opinion leaders and section B on active ageing processes, its determinants and the environmental factors that affect adoption of active ageing process in the community.

**Key informant interview:** there were 8 key informant interviews in both urban and rural communities. Each key informant interview lasted for 30-60 minutes. Through the community development chairman, the opinion leaders were identified. Those identified for interview were given a code by the researcher from A-H. The qualitative data collected during key informant interview were transcribed from the audio recording and hand written notes for data analysis. These were severally reviewed; important statements and cues were described and coded. Common themes were classified to provide a framework to revealing respondents knowledge, level of physical activity, level of community participation/ social engagement, life-long learning and factors affecting active ageing determinants in the communities.

**Data analysis:** the questionnaire was thoroughly checked, entered and analysed using IBM SPSS version 20 statistical software. Findings were presented in frequency tables and cross tabulations to determine relationships between variables. Chi-square was used to evaluate test of significance for categorical variables and t-test for continuous variables. The level of significance was set at p < 0.05.

The knowledge of respondents on active ageing processes and its determinants were graded based on the individual score. There were 10 knowledge questions. Each correct response was scored one and wrong response was scored zero. The total score was summed and graded as poor knowledge for score of 1-3, 4-6 for fair knowledge and = 7 as good knowledge [12]. The occupation of respondents were classified in hierarchical order according to the international standard classification of occupation following their level of education and training by international labour organization [13].

The physical activity of respondents measured the total time spent in various physical activities and the frequency of the physical activity in a week. Throughout the week, including activity for work, during transport to and from places and leisure time, adult should do at least 150 minutes of moderate intensity physical activity or 75 minutes of vigorous intensity physical activity for at least 3 days. Respondent who participates in any form of physical activity for 150 minutes of moderate intensity physical activity or 75 minutes of vigorous intensity physical activity or above for more than 3 days a week was graded as good physical activity while respondents with less than 150 minutes of moderate intensity physical activity or less than 75 minutes of vigorous intensity physical activity and less than 3 days a week was graded as poor physical activity [10]. The participation in community activities of respondents were focused on 9 community participation questions. Each question was scored into 1- 3 point Likert scale and the total score obtained by each respondent was graded as poor when the score was (< 9), fair when the score was (10-18), good when participation in community activity was greater than (> 18).

Lifelong learning of respondents was based on 5 lifelong learning questions. Each question was scored 1-3 point Likert scale and the total score obtained by each respondent was graded as poor with a score below (< 5), fair when the score of lifelong learning process was between (6-10) and good when lifelong learning was (>10) [14]. To ascertain the active ageing status of respondents in both urban and rural communities, the physical activity was re-coded. The score of 0 was assigned to number of days of physical activity less than 3 days and 1 to number of days 3 and above. Moderate intensity physical activity was re-coded as 0 for duration of the activity less than 150 minutes and 1 to duration 150 minutes and above. Also vigorous intensity physical activity was re-coded. The score of 0 was assigned to duration less than 75 minutes and 1 to 75 minutes and above. The range of score for the various domains of physical activity was 0-2. The range of score for community participation was 9-27 and that of life-long learning was 5-15. The various scores of the determinants were summed up and the median (m = 29) was determined. This was used to grade the active ageing of respondents into good active ageing process when greater than or equal to median and poor active ageing when less than median.

**Ethical approval:** it was obtained from Health research and ethical committee of Lagos University Teaching Hospital and oral informed consent was sought from the respondents before the interviewer administer the questionnaire.

## Results

### Socio-demographic characteristics

The mean age of respondents in the urban area was 48.6 ± 6.2 years while that of the respondents in rural area was 49.6 ± 6.3 years. The difference in the mean age was statistically significant (p < 0.05). Most of the respondents were of Yoruba ethnicity. There was a statistically significant difference in the ethnicity and religion among the respondents in the urban area and rural area (p < 0.05) (Table 1).

**Table 1:** socio-demographic characteristics of respondents

Variable	Urban N=360	Rural N=360	Total	P-value
**Age group (years)**	**Freq (%)**	**Freq (%)**		
40-44	111(30.8)	100(27.8)	211(29.3)	0.418
45-49	93(25.8)	86(23.9)	179(24.9)	
50-54	74(20.6)	69(19.2)	143(19.9)	
55-59 >60	66(18.3) 16(4.5)	83(23.1) 22(6.0)	149(20.4) 38(5.5)	
Mean ± SD	48.6 ± 6.2	49.6 ± 6.3	t=-2.026	0.043
**Ethnicity**				
Yoruba	181(50.3)	252(70.0)	433(60.1)	0.001
Igbo	114(31.7)	31(8.6)	145(20.1)	
Hausa	18(5.0)	15(4.2)	33(4.6)	
Others	47(13.0)	62(17.2)	109(15.2)	
**Marital status**				
Married	258(71.7)	311(86.4)	569(79.0)	0.001
Single	69(19.2)	18(5.0)	87(12.1)	
Divorced	12(3.3)	8(2.2)	20(2.8)	
Widower	8(2.2)	10(2.8)	18(2.5)	
Separated	13(3.6)	13(3.6)	26(3.6)	
**Education**				
No Formal Education	17(4.7)	59(16.4)	76(10.6)	0.001
Primary	44(12.2)	121(33.6)	165(22.9)	
Secondary	141(39.2)	124(34.4)	265(36.8)	
Tertiary	158(43.9)	56(15.6)	214(29.7)	
**Household income**				
<18000 Per month	21(5.8)	20(5.6)	41(5.7)	0.001
18000-35999	55(15.3)	118(32.8)	173(24.0)	
36000-53999	83(23.1)	88(24.4)	171(23.8)	
54000-71999	89(24.7)	88(24.4)	177(24.6)	
>72000	112(31.1)	46(12.8)	158(21.9)	
**Occupation**				
Unskilled	39(10.8)	64(17.8)	103(14.3)	
Semi-Skilled	138(38.3)	90(25.0)	228(31.7)	
Skilled	73(20.3)	175(48.6)	248(34.4)	
Professional	110(30.6)	31(8.6)	141(19.6)	

### Determinants of active ageing process

There was statistically significant difference in the level of physical activity, community participation and level of engagement in life-long learning among the respondents in urban area and rural area (p < 0.05). More of the respondents in the rural area 274(76.1%) practice good physical activity than the respondents in the urban area 204(56.7%). Also, higher number of the respondents in the rural area 251(69.7%) participate in community activities more regularly than the respondents in the urban area 204(56.7%). However, more respondents in the urban area 211(58.6%) engage in life-long learning activities more than the respondents in the rural area 201(55.8%) (Table 2). There was statistically significant difference in the status of active ageing process among the respondents in the urban area and rural area in this study (p < 0.05). Greater number of the respondents in the rural area 293(83.2%) than the respondents in the urban area 262(73.8%) were practicing good active ageing (Table 3).

**Table 2:** determinants of active ageing process among the urban and rural respondents

Determinants	Urban N=360	Rural N=360	Total	p-Value
**Level of physical activity**	**Freq (%)**	**Freq (%)**		
Poor	156(43.3)	86(23.9)	242	0.001
Good	204(56.7)	274(76.1)	478	
**Level of community participation**				
Poor	7(1.9)	0(0.0)	7	0.001*
Fair	149(41.4)	109(30.3)	258	
Good	204(56.7)	251(69.7)	455	
**Level of engagement in life-long learning**				
Poor	7(1.90	0(0.0)	7	0.012*
Fair	142(39.4)	159(44.2)	301	
Good	211(58.6)	201(55.8)	412	
*Fisher´s Exact Test				

**Table 3:** the status of active ageing process of respondents

Variable	Urban N=360	Rural N=360	Total	p-Value
	Freq (%)	Freq (%)		
Poor	93(26.2)	60(16.8)	153	0.002
Good	262(73.8)	293(83.2)	560	

### Factors associated with active ageing

The age of respondents in both the urban area and rural area was not significantly associated with active ageing practice. However, the ethnicity of respondents was significantly associated with the level of active ageing process in both urban and rural areas. There was statistically significant association between education of respondents in urban area and rural area with active ageing practice (p < 0.05). More respondents in the urban area with higher education than the rural counterpart practice good active ageing. However, education was not a factor for the adoption of active ageing practices among the rural respondents. More professionals in the urban area practice good active ageing than the professionals in the rural area while highly skilled workers in the rural area practice good active ageing more than the urban counterpart (Table 4). Multivariate logistic regression showed that physical activity (aOR 7.62, 95% CI: 243-23.94, P = 0.001), life-long learning (aOR 51.73, 95% CI: 12.14-220.49, P = 0.000) and community participation (aOR 3.46, 95% CI: 2.51-4.77, P = 0.000) are predictors of active ageing (Table 5).

**Table 4:** association between socio-demographic characteristics of respondents and level of active ageing

Variable	Urban (360)		Rural (360)	
Age group	Poor	Good	Poor	Good
40-44	34(31.2)	75(68.8)	14(14.3)	84(85.7)
45-49	24(26.1)	68(73.9)	16(18.6)	70(81.4)
50-54	14(18.9)	60(81.1)	13(18.8)	56(81.2)
55-59	20(31.2)	44(68.8)	13(15.7)	70(84.3)
60 and above	1(6.2)	15(93.8)	4(18.2)	18(81.8)
**TOTAL**	93	262	60	298
	P = 0.104*		P = 0.901*	
**Ethnicity**				
Yoruba	13(19.2)	143(80.8)	23(9.2)	228(90.8)
Igbo	35(31.0)	78(69.0)	11(35.5)	20(64.5)
Hausa	9(50.0)	9(50.0)	5(33.3)	10(66.7)
Others	15(31.9)	32(68.1)	21(34.4)	40(65.6)
TOTAL	93	262	60	298
	**P = 0.008**		**P = 0.001**	
**Marital status**				
Married	57(22.5)	196(77.5)	50(16.1)	260(83.9)
Single	25(36.2)	44(63.8)	5(29.4)	12(70.6)
Divorced	2(16.7)	10(83.3)	0(0.0)	8(100)
Widower	4(50.0)	4(50.0)	1(10.0)	9(90.0)
Separated	5(38.5)	8(61.5)	4(30.8)	9(69.2)
Total	93	262	60	298
	P = 0.046*		P = 0.215*	
**Education**				
No formal education	6(35.3)	11(64.7)	13(22.0)	46(78.0)
Primary	20(45.5)	24(54.5)	30(24.8)	91(75.2)
Secondary	42(30.7)	95(69.3)	14(11.4)	109(88.6)
Tertiary	25(15.9)	132(84.1)	3(5.5)	52(94.5)
**Total**	93	262	60	298
	**P = 0.001**		P = 0.002*	
**Occupation**				
Unskilled	19(51.4)	18(48.6)	16(25.0)	48(75.0)
Semi-skilled	45(33.1)	91(66.9)	17(19.1)	72(80.9)
Skilled	16(21.9)	57(78.1)	24(13.8)	150(86.2)
Professional	13(11.9)	96(88.1)	3(9.7)	28(90.3)
Total	93	262	60	298
	**P = 0.001**		P = 0.128*	

**Table 5:** univariable and multivariable predictors of active ageing

	Univariable analysis	Multivariable analysis		
	uOR (95% CI)	p-Value	aOR (95%CI)	p-Value
Age group	1.077(0.932 - 1.245)	0.314	0.959 (0.666 - 1.381)	0.823
Marital status	0.819(0.691 - 0.971)	0.021	1.440 (0.828 - 2.504)	0.196
Place of Residence	1.763 (1.224 - 2.538)	0.002	0.516 (0.170 - 1.561)	0.241
Level of Education	1.388(1.155 - 1.667)	0.000	0.777 (0.393 - 1.529)	0.462
Occupation	1.660(1.366 - 2.019)	0.000	1.152 (0.715 - 1.857)	0.561
Household Income	1.106(0.955 - 1.282)	0.180	0.756 (.492- 1.160)	0.200
Life Long learning	14.090(8.646 - 22.962)	0.000	51.731 (12.138-220.485)	0.000
Physical Activity	1.229(0.846 - 1.786)	0.280	7.621(2.426 - 23.943)	0.001
Community Participation	2.661(2.155 - 3.285)	0.000	3.455(2.505 - 4.766)	0.000

## Discussion

Active ageing process is usually determined by various factors and determinants like physical activity, social participation, continuous life-long learning and other socio-economic factors such as education and income that influences the adoption and practice of active ageing activities among individuals in the community. These are health promoting activities and individuals are at differing stages of adoption and practice. The composite influence of the individual activity determines how one is adopts and practice active ageing in the community. In this study, more of the respondents in the rural area (83.2%) adopts good active ageing practices than the respondents in the urban area (73.8%) (p < 0.05). This finding was in contrast to the study in Zambia on the determinants of active ageing where residents in the urban community appeared to participate more in the community activities than the rural residents (92.1% against 84.5%) and were likely to be more self-fulfilled (10.2% against 7.7%) than the rural residents [7, 15]. The age of respondents was not significantly associated with the level of active ageing process of the respondents. This therefore implies that age is not a barrier to the adoption of active ageing processes. Any one at any age can gradually adopt the active ageing processes in order to reduce the risks inherent in poor activity and in the sedentary lifestyle. This is one of the behavioural changes being espoused in order to reduce and begin to reverse the growing non-communicable diseases scourge in the population. Majority of the respondents in age groups 40-44 and 55-59 in the rural area compared to the respondents in the urban area were practicing good active ageing in their activities. Marital status was associated with active ageing among the married respondents in the urban area but not associated among the married respondents in rural area.

There was statistically significant association between ethnicity and active ageing in both urban and rural area (p < 0.05). More respondents of Yoruba extraction in the rural area practice good active ageing compared to the urban counterparts. This was corroborated during the key informant interview as majority of the respondents were from Yoruba ethnic group that are used to engage in one social event or the other most of the year in the community. One of the respondents during key informant interview in the rural community affirmed that there was usually social event in the community as it used to be an avenue to re-unite with family and friends. According to the respondents, *‘the community is not devoid of social life as we frequently have festivals such as masquerade festival, Asemo, Asa quarterly in a year in the community. This is a period of socialisation, rest from every day hustling and re-connection with both family and friends in the community.’*. Education was significantly associated with practice of active ageing (p < 0.05). More respondents in the urban area with tertiary education and are professionals practice good active ageing than the rural counterpart. Also, greater numbers of urban respondents who earn greater than #72000 practice good active ageing than the rural respondents who earn greater than #72000 in the community. These are indicators of level of socio-economic status of respondents in the communities. This was similar to the study in Zambia where socio-economic determinants such as education and monthly income were associated with good active ageing practice [7].

On the individual´s perception of their health with regards to active ageing, there was significant association between self-rated health of respondents and their level of active ageing process. More of the respondents in the rural area 80.3% than in the urban area 70.0% who rated their health to be good were practicing good active ageing process. Individuals and population groups should take some responsibilities to promote and protect health. These include being more active, avoid sedentary behaviour, engage in community activities, engage in learning and adopt healthy behavioural lifestyle. These are practices that studies have shown to reduce the risk of developing non-communicable diseases among the populace. The strength of the study is that it assessed active ageing process and its determinants using mixed method study and the limitation is misreporting and recall bias as data collection was by self- reporting. Casual inference cannot be made as it was cross sectional study in nature.

## Conclusion

The study examined the knowledge and determinants of active ageing to improve quality of life among men in both rural and urban communities. The findings suggest that rural dwellers adopt community practices that improve their quality of life than the urban dwellers even-though necessary support are needed in the rural areas to enable them maintain these practices. The respondents in the rural area were more regularly involved in the community activities as it is regarded as part of their social life and they derive a lot of satisfaction, a sense of dignity and self-esteem in those practices than the respondents in the urban area. Although, men in urban areas were involved more in life-long learning practices, it is not enough to improve quality of life as composite community practices are needed to ensure improved health outcomes. The urban community needs continuous public education and supportive environment to be able to easily adopt practices to improve quality of life and reduce the risk of non-communicable diseases among men in Nigeria. The value one attaches to health determines choices to improve health. In addition, age friendly and socially supportive environment influences healthy choices and adoption of community practices that enhances active ageing in population groups.

### What is known about this topic

- What is known on this topic has been the fact that physical activity, socio-economic factors determines the quality of life of individuals in the community;
- Active ageing was being espoused for the older age group to help reduce the risk of diseases and dependency.

### What this study adds

- What this study has added is that in other to improve the quality of life of individuals in the community, apart from physical activity and socio-economic factors, community participation and continuous learning contribute to improving the quality of life, disease prevention and improves sense of fulfilment among individuals;
- It has also brought that active ageing processes could be adopted at any age to promote healthy behaviours to improve health.
